# Single-cell profiling reveals GABBR1 as a cuproptosis-regulated prognostic biomarker in pancreatic cancer

**DOI:** 10.1016/j.iliver.2026.100276

**Published:** 2026-10-15

**Authors:** Jie Gong, Weinan Zhang, Li Tang, Yuxi Long, Xianxing Wang, Jiali Yang, Yongjun Yang, Roberto Montalti, Roberto Ivan Troisi, Huaizhi Wang

**Affiliations:** a University of Chinese Academy of Sciences (UCAS) Chongqing School, Chongqing Medical University, Chongqing 400016, China; b Chongqing School, University of Chinese Academy of Sciences, Chongqing 400714, China; c Institute of Hepatopancreatobiliary Surgery, Chongqing General Hospital, University of Chinese Academy of Sciences, Chongqing 401147, China; d Chongqing General Hospital, Key Laboratory of Intelligent Medicine Engineering for Hepatopancreatobiliary Diseases, Chongqing 401147, China; e Center of Urology and Nephrology, The Second Affiliated Hospital of Chongqing Medical University, Chongqing 401336, China; f Institute of Hepatopancreatobiliary Surgery, Chongqing General Hospital, Chongqing University, Chongqing 401147, China; g Department of Clinical Medicine and Surgery, Federico II University Hospital, Naples 80131, Italy

**Keywords:** Pancreatic cancer, Cuproptosis, *GABBR1*, Immune infiltration, Immunohistochemistry

## Abstract

**Background and aims:**

Cuproptosis is a recently identified copper-dependent form of cell death with potential therapeutic applications. However, its activity in cancer-associated fibroblasts (CAFs) and its association with pancreatic cancer (PC) prognosis remain poorly understood. This study aimed to address this gap.

**Methods:**

We performed integrated analyses of scRNA-seq data from patients with PC (GSE212966) and The Cancer Genome Atlas-pancreatic adenocarcinoma dataset. Based on cuproptosis-related AUCell scores, fibroblasts were stratified into high-score (cuproptosis-high) and low-score (cuproptosis-low) groups. Weighted gene coexpression network analysis (WGCNA) was used to identify modules associated with cuproptosis-related genes (CRGs), followed by random survival forest modeling to screen prognostic genes. Functional assessments included gene set variation analysis, immune infiltration analysis via CIBERSORT, and immunohistochemistry for experimental validation.

**Results:**

scRNA-seq analysis identified fibroblasts as the principal cell type with elevated cuproptosis activity. By integrating WGCNA with survival analysis, we identified *GABBR1* as a key gene whose expression decreased with increase in tumor progression. Fibroblasts expressing higher *GABBR1* levels also exhibited increased expression of immune checkpoint genes and T-cell activation markers. A nomogram integrating *GABBR1* expression with clinical features showed strong prognostic performance. Immunohistochemical validation confirmed that *GABBR1*, *LIAS*, and *LIPT1* were expressed at significantly lower levels in tumor tissues than in adjacent normal tissues. Importantly, *GABBR1* expression negatively correlated with PC progression and was linked to CRGs (*LIAS* and *LIPT1*), emerging as a novel CAF-specific biomarker.

**Conclusion:**

Modulating *GABBR1* can reshape cuproptosis pathways, thereby influencing apoptotic sensitivity and modulating immune responses.

**Table:** Table of Abbreviations

AUC	Area under the curve
CAF	Cancer-associated fibroblast
CRG	Cuproptosis-related gene
EMT	Epithelial–mesenchymal transition
GABA	Gamma-aminobutyric acid
GSEA	Gene set enrichment analysis
GSVA	Gene set variation analysis
PC	Pancreatic cancer
TOM	Topological overlap matrix
UMAP	Uniform manifold approximation and projection
WGCNA	Weighted gene coexpression network analysis

## 1 Introduction

Pancreatic cancer (PC) is one of the most aggressive malignancies worldwide. Its 5-year survival rate is approximately 10%, primarily because of late diagnosis, rapid progression, and resistance to standard therapies.^1^ Across both sexes, PC is the third leading cause of cancer-related death, and its incidence and mortality continue to increase each year.^2^ Despite advances in surgery, chemotherapy, and immunotherapy, outcomes for patients with PC remain poor, with approximately half presenting with metastatic disease at diagnosis.^1^, ^3^, ^4^, ^5^ These challenges underscore the urgent need to identify new biomarkers and therapeutic targets for this high-risk population.

The PC tumor microenvironment (TME) is characterized by dense desmoplastic stroma and continuous cross-talk between cancer cells and surrounding stromal components. This environment promotes tumor invasion, dissemination, and therapeutic resistance.^6^, ^7^, ^8^ Desmoplastic stroma can account for up to 90% of the tumor volume, physically limiting drug penetration while promoting tumor progression through biochemical signaling.^9^, ^10^, ^11^ Among stromal cells, cancer-associated fibroblasts (CAFs) are key mediators of TME remodeling, promoting immune suppression, angiogenesis, and extracellular matrix stiffening,^12^, ^13^, ^14^ all of which contribute to immune evasion and metastatic growth. Furthermore, single-cell studies have revealed functional heterogeneity among CAFs, including inflammatory CAFs, myofibroblastic CAFs, and antigen-presenting CAFs (apCAFs), each with specialized roles in tumor progression.^15^, ^16^ Therefore, CAFs represent promising therapeutic targets in PC.

Copper is an essential trace element that modulates numerous cellular processes.^17^, ^18^ Recently, cuproptosis has been proposed as a copper-dependent cell death mechanism linked to mitochondrial dysfunction that can occur in tumor cells.^19^, ^20^ Considering their therapeutic potential, cuproptosis and copper-based treatments have been extensively investigated in PC.^21^, ^22^ Copper-based therapies can suppress tumor growth and represent potential options for treating chemotherapy-resistant tumors.^21^ However, their roles in CAFs, which are key components of the PC TME, remain poorly characterized.

In this study, we analyzed single-cell RNA-seq data from patients with PC and annotated multiple cell types, including fibroblasts and T cells. Based on AUCell analysis of cuproptosis-related genes (CRGs), we categorized fibroblasts into high- and low-cuproptosis-AUCell subgroups (cuproptosis-high and cuproptosis-low, respectively) and screened for differentially expressed genes (DEGs) between the groups. We identified *GABBR1* as a critical gene in fibroblasts whose expression is inversely correlated with tumor progression. Furthermore, our findings revealed a correlation between *GABBR1* and CRGs (*LIAS* and *LIPT1*), suggesting that *GABBR1* can serve as a CAF-specific therapeutic target for PC. Overall, this study provides novel insights into CAF biology in PC and its relationship with cuproptosis, supporting further investigation of PC treatments and combination strategies involving copper-based agents.

## 2 Material and methods

### 2.1 Ethics approval

All procedures involving human participants were conducted in accordance with the ethical standards of the Declaration of Helsinki. The study was approved by the Ethics Committee of Chongqing General Hospital (approval no., KY S2022-080-01; approval date, December 30, 2022). Before enrollment, written informed consent was obtained from each participant or their legal guardian.

### 2.2 Acquisition and processing of pancreatic adenocarcinoma cohort data

Transcriptomic profiles, copy number variation data, and single-nucleotide polymorphism data for pancreatic adenocarcinoma (PAAD) were obtained from The Cancer Genome Atlas (TCGA). The dataset comprised 183 TCGA-PAAD specimens, including 179 tumor tissues and 4 nontumor controls.

Single-cell RNA-seq data (GSE212966) were downloaded from the Gene Expression Omnibus and comprised 6 normal and 6 tumor samples. After excluding low-abundance transcripts, we processed the expression matrix using the “Seurat” R package. The analytical pipeline included normalization, scaling, principal component analysis, batch correction with Harmony, and uniform manifold approximation and projection (UMAP) for dimensionality reduction. The “ElbowPlot” function was used to select the optimal number of principal components, and UMAP was used to visualize the distribution of distinct cell clusters. Subtype-specific marker genes were identified by setting the logfc.threshold parameter to 0.25 in the “FindAllMarkers” function. After quality control, 9 samples (3 normal and 6 tumor) were retained for downstream analysis.

### 2.3 AUCell

We used the “AUCell” R package to assess CRG activity across individual cell lineages in the scRNA-seq dataset. After visualization with “ggplot2,” cells were stratified into cuproptosis-high and cuproptosis-low fibroblast subgroups based on the median area under the curve (AUC) score. CRG activity was quantified in individual cells using AUCell. Within the fibroblast population, cells with AUCell scores greater than or equal to the median were assigned to the cuproptosis-high subgroup, whereas cells with AUCell scores below the median were assigned to the cuproptosis-low subgroup. Therefore, the cutoff was defined at the fibroblast-cell level, not at the TCGA sample level.

### 2.4 Weighted gene coexpression network analysis (WGCNA) for cuproptosis-related module

A gene coexpression network was constructed for all genes in the TCGA-PAAD dataset using the “WGCNA” R package. The 5000 genes with the highest variance in expression were selected for downstream analysis, with the soft threshold set to 7. To evaluate network connectivity, the resulting weighted adjacency matrix was transformed into a topological overlap matrix, from which a hierarchical clustering tree was generated. Distinct branches of the tree represented separate gene modules, each of which is assigned a unique color. Genes were grouped by expression similarity using weighted correlation coefficients, with coexpressed genes assigned to the same module. Cuproptosis levels for each patient in TCGA-PAAD were quantified using the “ssGSEA” package in R.

### 2.5 Random survival forest analysis

Feature genes were selected using the random survival forest algorithm implemented in the “randomForestSRC” R package. Prognosis-associated genes were ranked by importance using Monte Carlo simulation with 1000 iterations (nrep = 1000). Genes with relative importance values > 0.3 were retained as the final feature set.

### 2.6 Analysis of immune infiltration and response to immunotherapy

The CIBERSORT algorithm—a widely used tool based on support vector regression—was used to deconvolute immune cell subtype expression profiles within the TME. This approach uses 547 signature genes to identify 22 distinct human immune cell phenotypes, including various T-cell, B-cell, plasma cell, and myeloid subsets. We applied CIBERSORT to the TCGA-PAAD dataset to estimate the relative abundances of these 22 immune-infiltrating cell populations and perform correlation analyses between gene expression levels and immune cell composition.

Immunotherapy response was evaluated using the TIDE database with datasets from TCGA-PAAD, TCGA-PRAD, and TCGA-CESC.

### 2.7 Cell communication analysis

We reconstructed cellular interaction networks using the “CellChat” R package, with the built-in CellChatDB as the ligand–receptor reference. Significant ligand–receptor pairs were identified based on overexpressed ligands or receptors, with statistical significance set at *p* < 0.01. Differences in signaling across cell types and specific ligand–receptor interactions were visualized using the “netAnalysis_signalingRole_heatmap” and “netVisual_bubble” functions, respectively.

### 2.8 Functional analysis

PAAD samples were divided into high- and low-expression groups according to the expression levels of key genes. Gene set enrichment analysis (GSEA) was performed to compare the signaling pathway enrichment between the two groups. Annotated gene sets for subtype-specific pathways were obtained from the MsigDB database (version 7.0). Gene sets with an adjusted *p* < 0.05 were considered enriched and ranked according to the consistency score.

### 2.9 Statistical analysis

Statistical analyses were performed using SPSS 23.0 (IBM, USA), and graphs were plotted using GraphPad Prism 8.0.1 (GraphPad, USA). For comparisons of measurement data between two groups, Student's *t*-test was used when variances were homogeneous; otherwise, the Mann–Whitney *U* test was used. Count data were compared using the chi-square test or Pearson precision test. Survival was analyzed using the Kaplan–Meier (KM) method. Statistical significance was defined as *p* < 0.05.

## 3 Results

### 3.1 scRNA profiling of PC

Using UMAP dimensionality reduction, we classified all cells into 12 distinct clusters and visualized their distribution (Fig. 1A and B). The 12 clusters were identified based on the top three DEGs of known cell lineages, and their expression levels are shown in Fig. 1C. Next, CRG activity was determined for each cell using the “AUCell” R package. Higher CRG expression corresponded to higher AUCell values, which were visualized on UMAP plots. Cells with high AUCell values were predominantly located in the fibroblast population (Fig. 1D). Furthermore, we compared CRG AUCell scores across different cell clusters between PC tissues (tumor group) and adjacent nontumor tissues (normal group). Compared with normal tissues, tumor tissues showed significantly higher cuproptosis activity in fibroblasts, whereas no significant between-group difference was observed in T cells (Fig. 1D). To distinguish cell populations, we performed differential expression analysis across cell types and highlighted the five most strongly upregulated and downregulated genes (Fig. 1E).

**Fig. 1:**
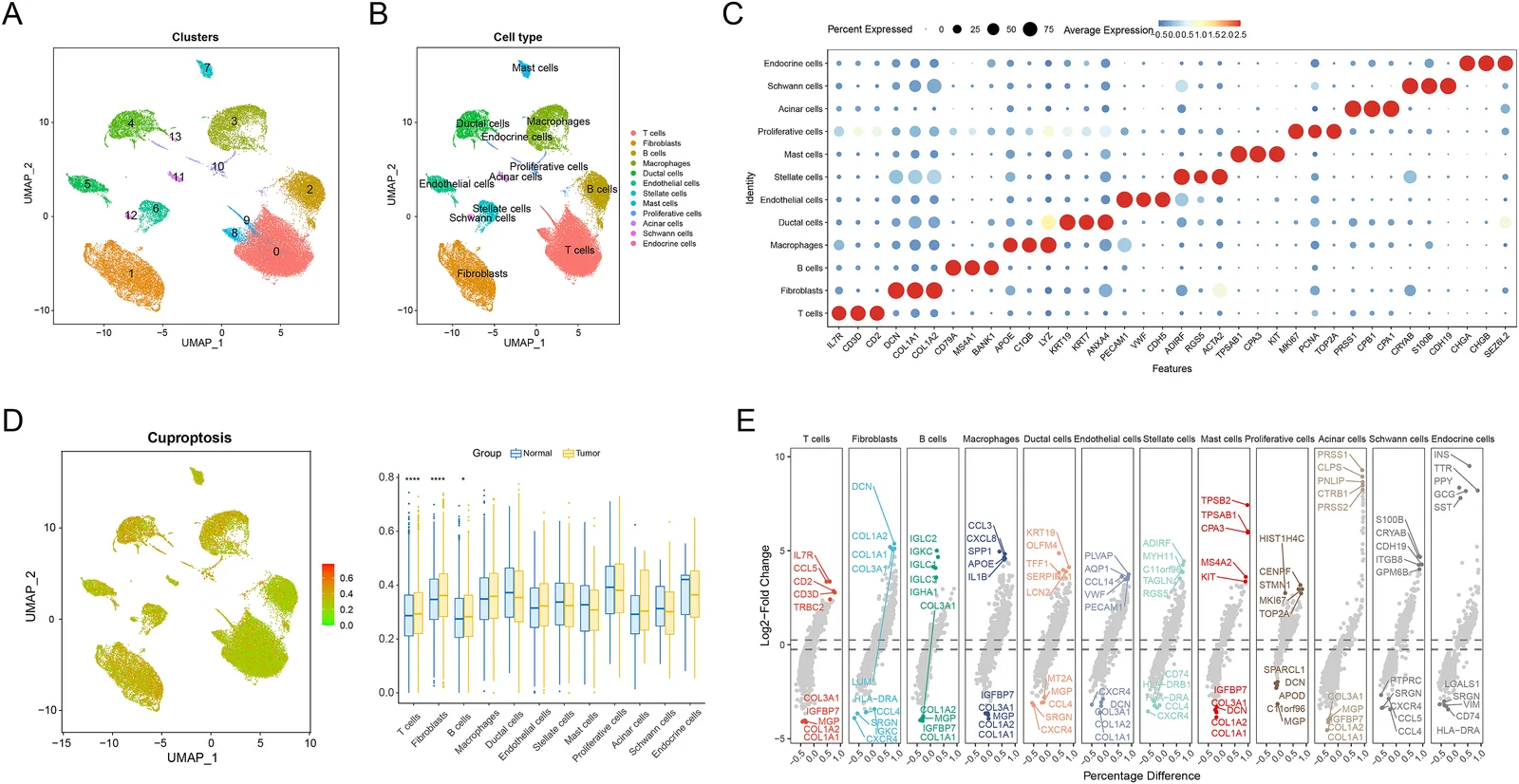
**Annotation of single-cell data and cuproptosis activity.** (A) Uniform manifold approximation and projection (UMAP) plot depicting all cells. (B) UMAP plot showing the annotation of all cells into 12 cell types. (C) Dot plots showing the expression of the top three differentially expressed genes for each cell type. (D) AUCell values indicating cuproptosis activity in each cell combined with a box plot of AUCell values from different cell clusters in the pancreatic cancer (PC; tumor) and adjacent tissue (normal) groups. (E) Top five up- and downregulated genes in different cell types. ∗*p* < 0.05 and ∗∗∗∗*p* < 0.0001.

### 3.2 Identification of the most relevant genes involved in cuproptosis activity

Because CAFs are major regulators of TME remodeling, we investigated fibroblasts in greater detail. To investigate the influence of CRGs on fibroblasts, we assigned each fibroblast a CRG-based AUCell score and divided the cells into cuproptosis-high and cuproptosis-low groups using the median AUCell score. To characterize pathway differences between these subgroups, we performed gene set variation analysis (GSVA). The top five pathways enriched in the cuproptosis-high fibroblast group included oxidative phosphorylation, protein secretion, epithelial–mesenchymal transition (EMT), fatty acid metabolism, and MYC target V1 (Fig. 2A). Several metabolic and biosynthetic pathways, including oxidative phosphorylation, protein secretion, fatty acid metabolism, and glycolysis, were significantly enriched in this subgroup. Marked activation was observed in key regulatory pathways, including MYC target V1, PI3K/AKT/mTOR signaling, and DNA repair. In addition, the enrichment of processes related to cell structure and function, including EMT and apical junctions, reflected the active role of cuproptosis-high fibroblasts in tissue remodeling. The mitotic spindle, unfolded protein response, TGF-β signaling, and hypoxia pathways were also upregulated in this group. Collectively, these pathways suggest that fibroblasts play an important role in cell proliferation, tumor metastasis, and adaptation to hypoxic environments.

**Fig. 2:**
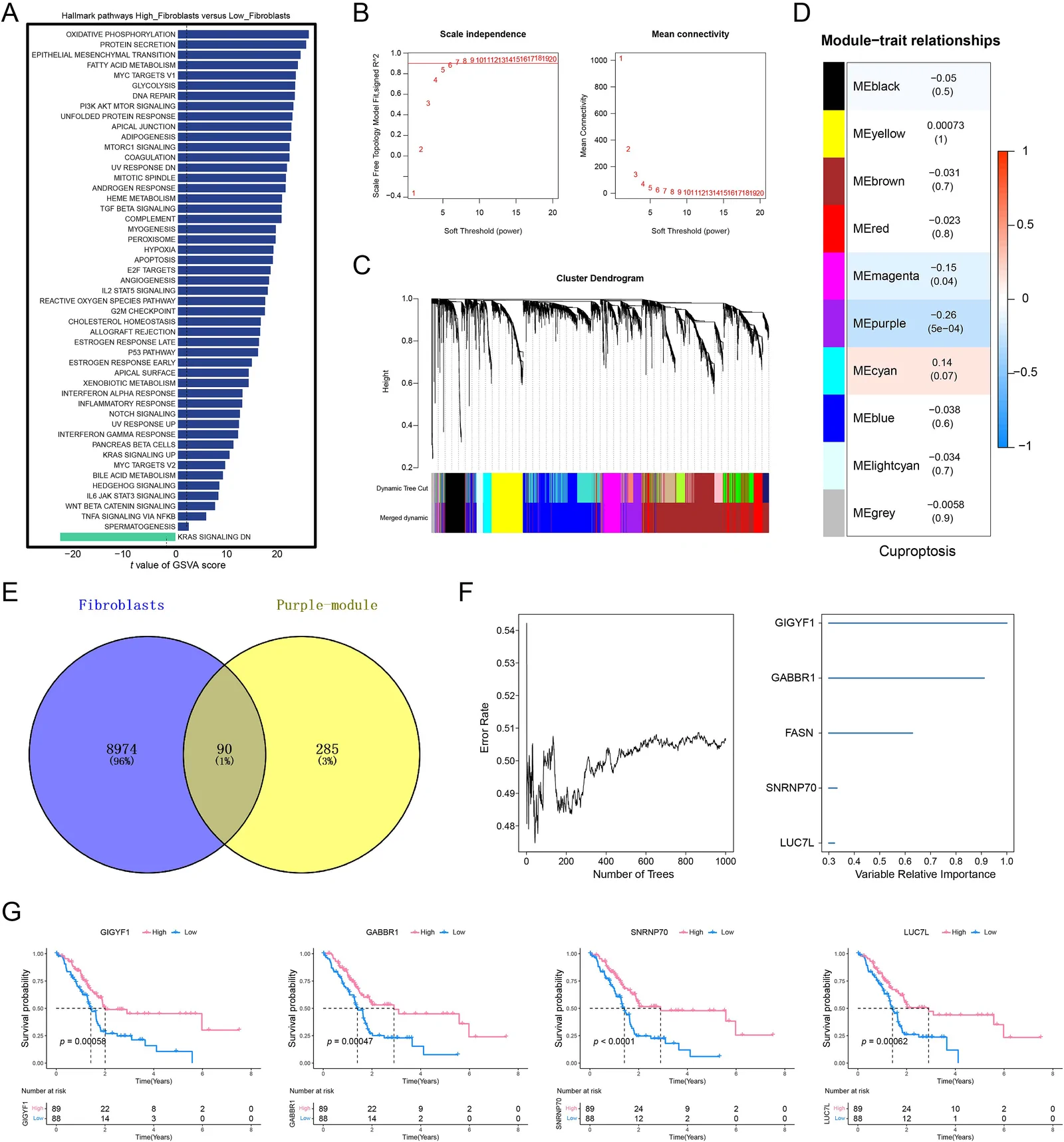
**Construction of cuproptosis-related prognostic model.** (A) Gene set variation analysis (GSVA) showing the enrichment of hallmark gene sets comparing cuproptosis-high and cuproptosis-low fibroblast groups. (B) Weighted gene coexpression network analysis (WGCNA). At a soft-thresholding value of 7, the mean connectivity tended to stabilize. (C, D) WGCNA identification of the modules most strongly associated with cuproptosis activity. (E) Venn diagrams showing the genes most associated with cuproptosis activity in fibroblasts. (F) Random forest algorithm was used to select key genes. (G) Survival probability curves of four key genes (*GIGYF1*, *GABBR1*, *SNRNP70*, and *LUC7L*) in patients with pancreatic cancer (PC).

Using ssGSEA, we assigned a cuproptosis score to each sample in the bulk RNA-seq dataset and subsequently performed WGCNA to identify gene sets that covaried with the cuproptosis score. With the soft-thresholding power set to 7, the mean connectivity tended to stabilize (Fig. 2B) and the data more closely approximated a power-law distribution, supporting their suitability for further analysis. After setting the minimum number of module genes to 100, deepSplit to 3, and merged modules with similarity <0.25, a total of nine nongray modules were identified (Fig. 2C and D). Subsequently, we evaluated the association between the expression of each module and clinical characteristics. Finally, we intersected 8974 DEGs between the cuproptosis-high and cuproptosis-low fibroblast groups with the purple module genes, which are most negatively associated with cuproptosis activity from WGCNA (Fig. 2E). The intersection yielded 90 overlapping genes that were selected for further analysis (Fig. 2E). Using these 90 genes, we constructed a random survival forest model and identified five characteristic genes with relative importance > 0.3: *GIGYF1*, *GABBR1*, *FASN*, *SNRNP70*, and *LUC7L* (Fig. 2F). Next, we evaluated the ability of these five cuproptosis-related DEGs to predict survival in patients with PC. Survival probability analysis demonstrated that higher expression of *GIGYF1*, *GABBR1*, *SNRNP70*, and *LUC7L* was associated with a better prognosis (Fig. 2G).

### 3.3 Construction of prognostic nomograms integrating clinical characteristics with specific cuproptosis-related DEGs

We constructed a prognostic nomogram based on clinical characteristics (i.e., age, sex, grade, and stage) and the expression levels of the selected genes to predict 1- and 3-year survival probabilities in patients with PC (Fig. 3A). Each variable was assigned a specific score, and the total score was used to predict survival probabilities. The calibration curve of the nomogram showed good agreement between the predicted and observed OS for both 1- and 3-year survival (Fig. 3B). Next, we performed receiver operating characteristic analysis to evaluate the predictive performance of the model. The AUC was 0.647 for 1-year survival prediction and 0.796 for 3-year survival prediction, indicating good predictive accuracy over time (Fig. 3C). A heatmap was generated to visualize the correlations between the prognostic signatures, clinical characteristics, and the expression levels of selected genes (Fig. 3D), indicating that high expression of the selected genes was closely associated with a favorable prognosis in patients with PC. Decision curve analysis further showed that the selected gene expression levels provided the greatest net benefit for prediction (Fig. 3E).

**Fig. 3:**
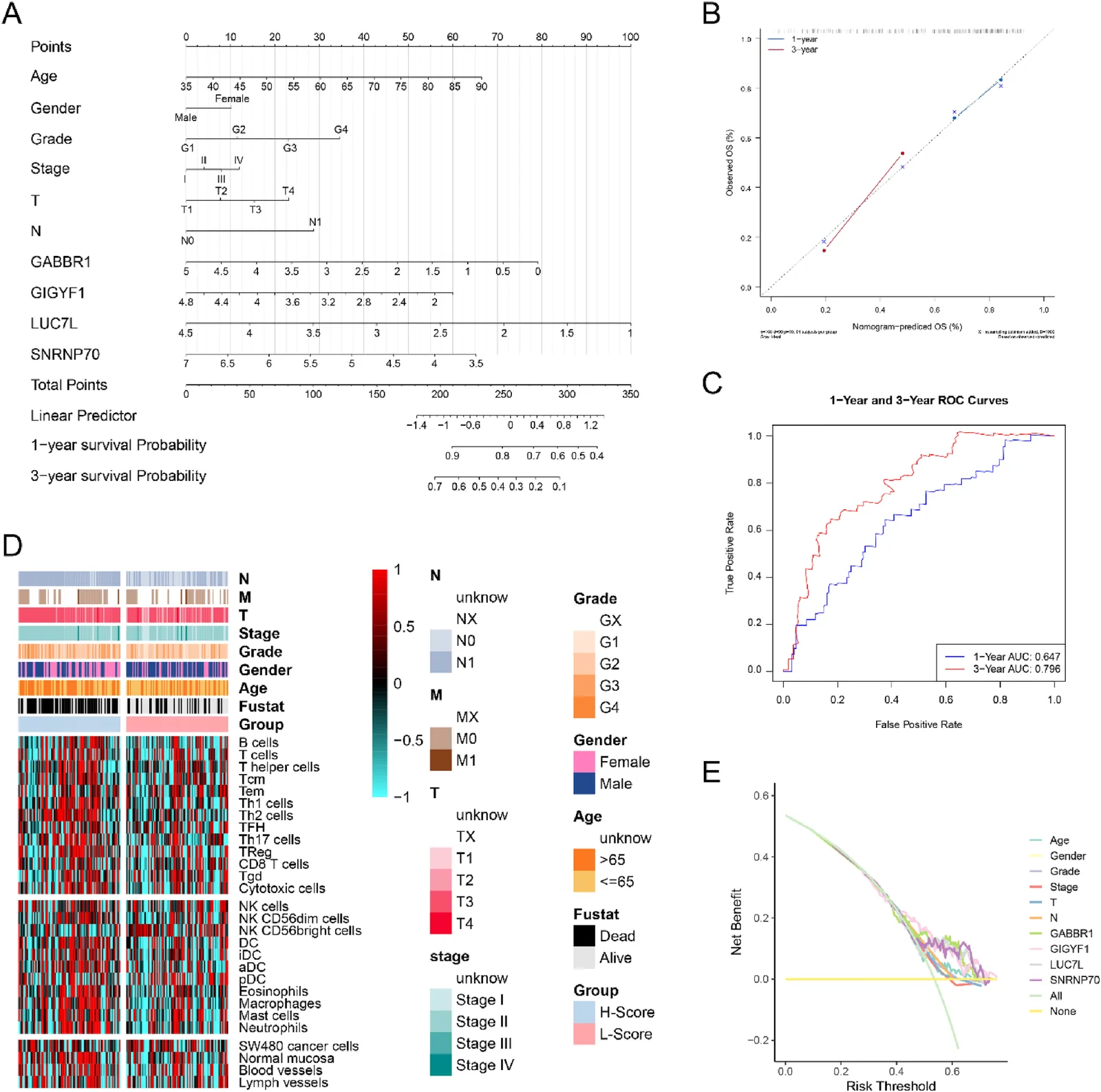
**Construction of nomogram**. (A) A nomogram was constructed by combining risk scores and clinical characteristics. (B) Nomogram calibration curves for the signature at 1 and 3 years. (C) Receiver operating characteristic curves for the signature at 1- (95% confidence interval: 0.658–0.766) and 3-year time points. (D) Clinical heat map incorporating model genes, clinical characteristics, and immune cells. (E) Decision curve analysis for different genes.

### 3.4 Correlation between GABBR1 expression of fibroblasts and tumors

We visualized the spatial expression patterns of four characteristic cuproptosis-related DEGs (*GABBR1*, *GIGYF1*, *SNRNP70*, and *LUC7L*) in PC cells using UMAP plots (Fig. 4A), indicating that each gene had a distinct expression profile across different cells. Violin plots showed the distribution of gene expression levels across various cell types (Fig. 4A), suggesting that *GABBR1* is more abundant in fibroblasts than in other cell types. To characterize *GABBR1*-associated pathway differences in fibroblasts, we performed GSVA to compare *GABBR1-high* fibroblasts (*GABBR1* H) with *GABBR1-low* fibroblasts (*GABBR1* L). GSVA revealed upregulated hallmark pathways including MYC targets, mTORC1 signaling, and EMT, indicating that *GABBR1* may regulate critical pathways associated with tumor progression and TME (Fig. 4B). Somatic mutation analysis was performed to characterize the distribution of genetic alterations in the *GABBR1* H and *GABBR1* L groups (Fig. 4C). These alterations included missense mutations, frameshift deletions/insertions, nonsense mutations, and splice-site variations. Mutation burden did not differ significantly between the two groups. We also compared the expression of representative immune-related genes and immune checkpoint-related genes between the *GABBR1* H and *GABBR1* L fibroblast groups (Fig. 4D). Several immune-related genes, including *ADORA2A*, *CD160*, *CD247*, *CD4*0LG, *CTLA4*, *ICOS*, *IDO2*, *PDCD1*, *TLR9*, and *TNFRSF4*, were significantly more highly expressed in the *GABBR1* H group than in the *GABBR1* L group. These genes participate in immune regulation, T-cell activation, and inflammatory signaling. In contrast, *FGL1* was significantly downregulated in the *GABBR1* H group compared with that in the *GABBR1* L group (Fig. 4D). These findings suggest that *GABBR1* plays a role in immune response modulation. Furthermore, GSEA identified pathways enriched in the *GABBR1* L fibroblast group compared with the *GABBR1* H group, including abnormal glycosylation, abnormal circulating proteinogenic amino acid derivative concentration, and PAAD (Fig. 4E). GSEA findings suggest that *GABBR1-low* fibroblasts contribute to immunosuppression and tumor progression.

**Fig. 4:**
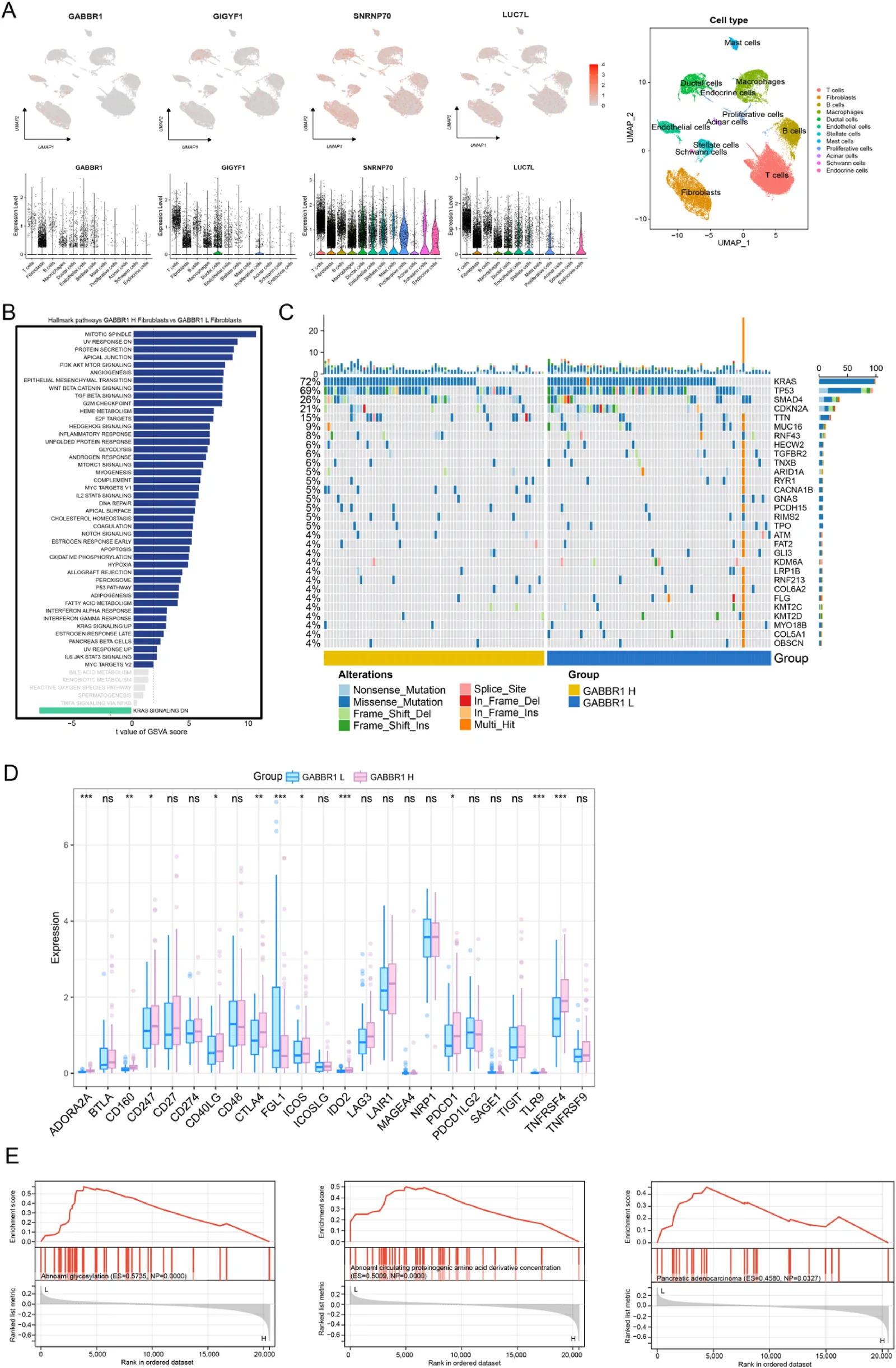
**Role of *GABBR1* expression in fibroblasts in tumors.** (A) Spatial patterns and violin plots for *GABBR1*, *GIGYF1*, *SNRNP70*, and *LUC7L* expression. (B) GSVA showing hallmark pathway enrichment in different risk subgroups. (C) Mutation landscape of the top 30 genes with the highest frequency of mutation in the *GABBR1* H and *GABBR1* L fibroblast groups. (D) Box plots of representative immune-related genes between the *GABBR1* H and *GABBR1* L fibroblast groups. (E) Gene set enrichment analysis of abnormal glycosylation, abnormal circulating proteinogenic amino acid derivative concentration, and pancreatic adenocarcinoma. ∗*p* < 0.05, ∗∗*p* < 0.01, ∗∗∗*p* < 0.001. ns, not significant.

### 3.5 Experimental validation of key genes

We stratified TCGA gene expression data into *GABBR1* H or *GABBR1* L groups and used these groups to identify *GABBR1-related* DEGs between the two groups. Notably, a heatmap showed that *LIAS* and *LIPT1* were significantly upregulated in the *GABBR1* H group compared with those in the *GABBR1* L group (Fig. 5A). Furthermore, correlation analysis revealed a moderate association between *GABBR1* and *LIAS* as well as between *GABBR1* and *LIPT1* (Fig. 5B). To assess the differential expression of *GABBR1*, *LIAS*, and *LIPT1*, we performed immunohistochemistry (IHC) assays (Fig. 5C), which showed significantly lower expression levels in tumor tissues of *GABBR1*, *LIAS*, and *LIPT1* than adjacent nontumor tissues (Fig. 5C).

**Fig. 5:**
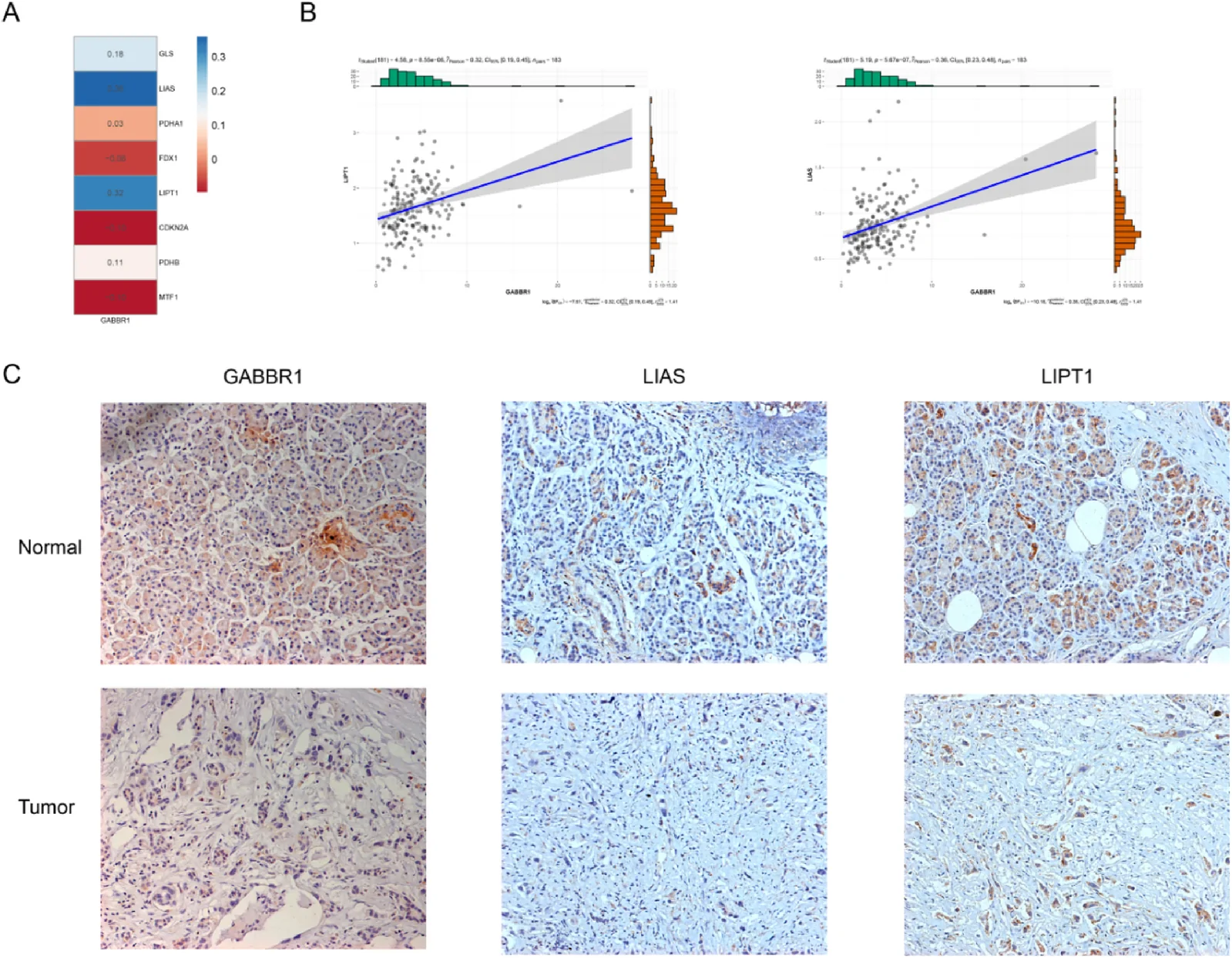
**Correlation among *GABBR1*, *LIPT1*, and *LIAS* expression.** (A) Heatmap of cuproptosis-related gene expression in *GABBR1* H and *GABBR1* L fibroblasts. (B) Correlation analysis of *GABBR1* with *LIAS* and *LIPT1*. (C) Immunohistochemical validation of *GABBR1*, *LIPT1*, and *LIAS* expression in normal and tumor tissues.

## 4 Discussion

PC remains one of the most aggressive solid tumors. Its TME is characterized by a dense desmoplastic stroma dominated by CAFs. These CAFs impede drug delivery and promote therapeutic evasion.^23^ Despite improvements in the 5-year survival rate of patients with PC from 1977 to 2019 following advances in multidisciplinary care and multimodal therapy, prognosis remains poor.^2^, ^24^

The discovery of regulated cell death mechanisms—particularly cuproptosis—has created new therapeutic opportunities.^25^, ^26^, ^27^ Excessive copper binding to lipoylated components of the tricarboxylic acid cycle can trigger cuproptosis.^19^, ^20^ Copper homeostasis has been reported to play an important role in tumor development and invasiveness, and the cuproptosis pathway represents a novel therapeutic target for PC.^22^, ^28^ Although CAFs are closely associated with PC progression,^12^, ^13^ studies on the relationship between CAF-specific cuproptosis and PC prognosis are limited.

Signature genes identified through single-cell transcriptomic analyses have been used to develop prognostic models targeting specific cell subsets.^29^, ^30^, ^31^ In the present study, we analyzed single-cell sequencing data from patients with PC and annotated various cell populations, including fibroblasts, T cells, and B cells. Subsequently, we used the cuproptosis gene set from the GeneCards database to calculate cuproptosis activity via the AUCell algorithm. High cuproptosis activity was observed in fibroblasts. Based on CRG AUCell values, we categorized single-cell populations into cuproptosis-high and cuproptosis-low groups, and fibroblasts in the tumor group had higher CRG AUCell values than those in the normal group. Pathways associated with tumor progression were significantly activated in the cuproptosis-high fibroblast subset. Oxidative phosphorylation plays a central role in cancer resistance and drug tolerance and holds potential as a therapeutic target.^32^, ^33^, ^34^ By enhancing tumor-initiating and metastatic potential, EMT functions as a key cellular program in malignant tumor progression.^35^, ^36^ Tumor aggressiveness and poor prognosis have also been associated with MYC target V1.^37^, ^38^ Metabolic and biosynthetic processes such as protein secretion, fatty acid metabolism, and glycolysis were upregulated in the cuproptosis-high fibroblast subset. Consistent with these findings, Alver et al. reported increased activity of invasive front pathways in CAFs, including MYC targets, glycolysis, and EMT.^39^ We hypothesize that these CAFs are closely associated with tumor invasion and metastasis. Collectively, these results indicate that fibroblast CRGs are potential prognostic factors in PC.

Prognostic models based on genes associated with specific biological processes have demonstrated robust predictive performances across various cancer types.^40^, ^41^ Based on the DEGs associated with fibroblast cuproptosis, five DEGs that differed between the two groups were significantly associated with prognosis. Of these, *GIGYF1*, *GABBR1*, *SNRNP70*, and *LUC7L* were selected to construct the prognostic model. Higher expression of these genes was associated with favorable prognosis in patients with PC. The *GABBR1* gene encodes gamma-aminobutyric acid (GABA) B receptor 1, and few studies have examined the association between *GABBR1* expression and cancer prognosis.^42^ Comparing *GABBR1*-high fibroblasts with *GABBR1-low* fibroblasts, *GABBR1-high* fibroblasts exhibited elevated expression of immune checkpoint genes (*CTLA4* and *PDCD1*) along with T-cell activation markers (*ICOS*), suggesting their ability to suppress and engage antitumor immunity. This bimodal immunomodulation is consistent with recent findings on apCAFs, which recruit regulatory T cells to suppress the immune response^43^ while promoting major histocompatibility complex class II immunity.^44^ These findings support further exploration of targeted agents in combination with immunotherapy. However, these findings were not confirmed using other techniques, and the underlying molecular mechanisms require further investigation.

Although targeting CAFs is considered a promising therapeutic approach, depleting CAFs may also promote tumor progression due to their functional heterogeneity.^45^, ^46^ Reprogramming active fibroblasts into quiescent fibroblasts is a novel strategy for inhibiting PC progression and improving the therapeutic response of cancer cells.^45^, ^46^ GSEA showed that *GABBR1-low* fibroblasts were associated with abnormal glycosylation, abnormal circulating proteinogenic amino acid derivative concentration, and PAAD, indicating immunosuppression and tumor progression. GABA has been implicated in mitochondrial function^47^; accordingly, we speculate that *GABBR1* maintains mitochondrial homeostasis in CAFs and participates in their reprogramming. In addition, *GABBR1* expression correlated with *LIAS* and *LIPT1*. Notably, deficiency in these CRGs (*LIAS* and *LIPT1*) confers resistance to cuproptosis.^20^ Compared with adjacent nontumor tissues, tumor tissues showed significantly lower expression of *GABBR1*, *LIAS*, and *LIPT1*. Decreased *GABBR1* expression in tumor tissues may reduce the capacity to induce cuproptosis, thereby creating a microenvironment conducive to tumor survival and suggesting *GABBR1* as a potential therapeutic target. Nevertheless, its expression in tumor-specific fibroblasts warrants further investigation.

This study has several limitations. First, the analyses were primarily based on public transcriptomic datasets, and the limited number of single-cell samples may restrict the generalizability of the findings. Second, AUCell scores reflect the enrichment of cuproptosis-related gene expression rather than directly measuring cuproptosis or cellular sensitivity to copper-induced death. The associations identified between GABBR1 expression, cuproptosis-related genes, and immune features therefore require functional validation. Third, although immunohistochemistry supported differences in protein expression between tumor and adjacent nontumor tissues, it did not establish the specific role of GABBR1 in CAFs. Further studies incorporating cell-type-specific localization, functional perturbation, and independent clinical cohorts are needed to clarify the underlying mechanisms and validate the prognostic utility of these findings.

## 5 Conclusion

Our findings clarify the key role of *GABBR1* in regulating cuproptosis activity within CAFs and its prognostic value in PC. Through integrated single-cell and bulk RNA-seq analyses, we identified *GABBR1* as a key gene inversely associated with tumor progression and linked to immune-related pathways and cuproptosis-associated mechanisms, including those involving *LIAS* and *LIPT1*. Experimental validation confirmed reduced *GABBR1* expression in tumor tissues, and its association with altered cuproptosis sensitivity. By incorporating *GABBR1* expression and clinical variables, the prognostic nomogram demonstrated robust predictive accuracy and potential translational value. These findings imply that targeting *GABBR1* may reprogram CAFs to enhance stromal vulnerability to copper-based therapies while synergizing with immunomodulatory strategies. However, additional mechanistic studies are needed to define the cross-talk between GABA signaling, cuproptosis sensitivity, and stromal–immune interactions.

The present study highlights the therapeutic potential of stroma-focused strategies for overcoming desmoplastic resistance in PC. Recent PC research has increasingly focused on stromal reprogramming and copper-dependent therapeutic approaches. The present study characterized cuproptosis in CAFs in PC, particularly the role of *GABBR1* in regulating cuproptosis activity and its prognostic significance. Future studies aimed at improving PC outcomes may explore combined strategies targeting both stromal and tumor cells.

## CRediT authorship contribution statement

**Jie Gong:** Writing – original draft, Visualization, Supervision, Software, Resources, Project administration, Methodology, Investigation, Data curation, Conceptualization. **Weinan Zhang:** Writing – original draft, Visualization, Supervision, Software, Resources, Project administration, Methodology, Investigation, Data curation, Conceptualization. **Li Tang:** Writing – original draft, Visualization, Supervision, Software, Resources, Project administration, Methodology, Investigation, Data curation, Conceptualization. **Yuxi Long:** Writing – original draft, Visualization, Supervision, Software, Resources, Project administration, Methodology, Investigation, Data curation, Conceptualization. **Xianxing Wang:** Validation, Formal analysis. **Jiali Yang:** Validation, Formal analysis. **Yongjun Yang:** Validation, Formal analysis. **Roberto Montalti:** Writing – review & editing, Writing – original draft, Visualization, Supervision, Software, Resources, Project administration, Methodology, Investigation, Data curation, Conceptualization. **Roberto Ivan Troisi:** Writing – review & editing, Writing – original draft, Visualization, Supervision, Software, Resources, Project administration, Methodology, Investigation, Data curation, Conceptualization. **Huaizhi Wang:** Writing – review & editing, Writing – original draft, Visualization, Supervision, Software, Resources, Project administration, Methodology, Investigation, Funding acquisition, Data curation, Conceptualization.

## Informed consent

Informed consent was obtained from all participants or their legal guardians included in the study.

## Ethics statement

This study was performed in accordance with the principles of the Declaration of Helsinki and was approved by the Ethics Committee of Chongqing General Hospital (approval no. KY S2022-080-01; approval date: December 30, 2022).

## Organ donation

Not applicable.

## Animal treatment

Not applicable.

## Data availability statement

All data supporting the findings of this study are available from the corresponding author upon reasonable request.

## Declaration of generative AI and AI-assisted technologies in the manuscript preparation process

During the preparation of this work, the authors used OpenAI solely for language polishing and grammatical improvement. After using this tool/service, the authors reviewed and edited the content as needed and take full responsibility for the content of the published article.

## Funding

This work was supported by the Chongqing Technology Innovation and Application Development Special Key Project [grant number CSTB2022TIAD-KPX0170].

## Declaration of competing interest

The authors declare no conflict of interest. The funders had no role in the design of the study; in the collection, analyses, or interpretation of data; in the writing of the manuscript, or in the decision to publish the results.
